# Chronic Δ9-Tetrahydrocannabinol Inhalation Following Osteoporosis Results in Bone Deficits

**DOI:** 10.3390/biomedicines14071620

**Published:** 2026-07-18

**Authors:** Aidan Powell, Grace Clouse, Samantha L. Penman, Isaiah T. Taylor, Faisal Sadar, Nihamul Ehan, Ayanna Varma, Michael Hadjiargyrou, David E. Komatsu, Panayotis K. Thanos

**Affiliations:** 1 Behavioral Neuropharmacology and Neuroimaging Laboratory on Addictions (BNNLA), Clinical Research Institute on Addictions, Department of Pharmacology and Toxicology, Jacobs School of Medicine and Biomedical Sciences, University at Buffalo, 1021 Main Street, Buffalo, NY 14203-1016, USA; 2 Department of Orthopaedics and Rehabilitation, Renaissance School of Medicine, Stony Brook University, Stony Brook, NY 11794-8181, USA; 3 Biological & Chemical Sciences, New York Institute of Technology, Old Westbury, NY 11568-8000, USA; mhadji@nyit.edu

**Keywords:** delta-9-tetrahydrocannabinol, osteoporosis, cannabis, bone, inhalation, ovariectomy

## Abstract

**Background**: Osteoporosis, a debilitating bone disease characterized by low bone mineral density, poses a large burden on the population. Current pharmacological treatment options are limited, with antiresorptive drugs being the current first-line option. Recent research has shown that the endocannabinoid system, modulated by endocannabinoids and phytocannabinoids, may influence bone remodeling. As the prevalence of cannabis use and research into its medicinal potential continues to increase, its therapeutic potential has emerged. **Objective**: To investigate the role of Δ9-tetrahydrocannabinol (THC) inhalation in treating osteoporosis in a rodent model. **Methods**: Adult female Sprague–Dawley rats underwent ovariectomy (OVX) to induce osteoporosis. Four weeks post-OVX, rats received either THC or Air treatment via inhalation for 8 weeks. A control group of age-matched rats received sham surgery. Rats were then euthanized at 38 weeks old, and hindlimb samples were collected for caliper, microCT, and biomechanical analyses. **Results**: OVX Air-treated rats showed significantly decreased trabecular bone volume, trabecular bone volume fraction, trabecular separation, trabecular tissue mineral density, trabecular number, and connective density compared to sham surgery controls. Compared with Air controls, OVX rats treated with THC showed increased endosteal volume (20%; *p* < 0.05). THC-treated rats also showed decreased trabecular bone mineral density (2%; *p* < 0.005) and trabecular thickness (14%; *p* < 0.05). **Conclusions**: Chronic inhaled THC worsened key aspects of trabecular bone microarchitecture. Results do not support use of THC vapor as a therapeutic agent for osteoporosis and suggest THC may adversely affect bone quality in estrogen-deficient states. Further research is needed to evaluate dose-dependent effects and to distinguish the skeletal impacts of different cannabinoid components.

## 1. Introduction

Osteoporosis, one of the most common metabolic bone diseases, is characterized by low bone mineral density and an elevated risk of fractures [1]. Osteoporotic fractures, especially in the hip and vertebrae, are associated with serious clinical consequences such as long-term disability, loss of independence, chronic pain, and high rates of morbidity and mortality [2,3,4]. Osteoporosis is especially common in post-menopausal women due to the sharp decline in estrogen [5], which is vital to maintaining skeletal homeostasis through the balanced activity of osteoclasts and osteoblasts leading to normal bone remodeling [6,7,8]. There are currently three major classes of drugs available to treat post-menopausal osteoporosis: antiresorptive agents, anabolic agents, and dual-action agents with both antiresorptive and anabolic properties [2]. Although these drugs are effective, a large percentage of patients are not offered appropriate treatment or resist treatment due to concern about adverse effects [2,9,10].

*Cannabis sativa*, commonly known as marijuana, is a plant that has been used recreationally and medicinally for centuries [11]. The main psychoactive constituent, Δ9-tetrahydrocannabinol (THC), exerts its biological effects through modulation of the endocannabinoid system [12,13,14]. The endocannabinoid system (ECS) is the endogenous system of receptors and agonists and the main site of action for many cannabinoids [15,16]. The ECS primarily comprises cannabinoid receptors 1 (CB1) and 2 (CB2), the transient receptor potential vanilloid 1 (TRPV1) channel, endogenous ligands anandamide (AEA) and 2-arachidonoylglycerol (2-AG), and respective degrading enzymes, such as fatty acid amide hydrolase (FAAH) and monoacylglycerol lipase (MAGL) [15,17,18,19]. THC is a partial agonist of both CB1 and CB2 receptors but notably has a greater affinity for CB1 [20]. THC offers a therapeutic potential for a multitude of neurological disorders through neuroregulatory, anxiolytic, anticonvulsant, and antispasmodic properties [21,22,23,24,25,26,27,28,29,30,31].

Bone cells, including osteoblasts and osteoclasts, express CB1, CB2, TRPV1, AEA, 2-AG, and synthesis/degradation enzymes, allowing them to modulate the ECS locally [32,33,34,35]. Endocannabinoids (eCBs), the neurotransmitters that bind to cannabinoid receptors, are involved in the coupled remodeling of bone, thereby modulating bone strength and mineral homeostasis via activation of osteoblasts and osteoclasts [36]. CB1 and TRPV1 activation has been shown to be osteoclastogenic and induce bone resorption, whereas CB2 is anti-osteoclastogenic and reduces resorption [34,37]. This balance of osteoclast and osteoblast activation and inhibition by CB1 and CB2 is a potential application in which eCB modulation via THC, CBD, or other cannabinoids may be critically important for osteoporosis and other degenerative bone diseases [38].

Despite increasing evidence supporting the role of cannabinoids in bone metabolism, there is a lack of data regarding the effects of chronic THC inhalation on bone health, particularly in the context of estrogen deficiency. Additionally, most existing research investigating the impact of cannabinoids on skeletal health has relied on intraperitoneal or oral administration routes rather than inhalation methods [39,40,41]. These forms of administration fail to accurately reflect the most common route of clinical cannabis consumption, smoking or vapor inhalation [40,41,42,43,44]. In both rodents [45] and humans [46], there are significant differences in THC pharmacokinetics depending on the route of administration, and inhalation provides significant advantages in preclinical studies in terms of translation. Although many preclinical studies have demonstrated that synthetic CB2-specific agonists have the ability to rescue ovariectomy-induced bone loss in rodent models, these results may not necessarily translate to inhaled whole-plant THC, whose effects on bone health remain largely understudied [47]. Moreover, clinical findings on cannabis use and bone health have been inconsistent: some studies suggest that chronic recreational cannabis use correlates with decreased bone mineral density (BMD) and higher fracture risk, while others highlight cannabinoid receptor modulation as a means of promoting osteogenesis and bone remodeling [48,49,50,51]. These conflicting outcomes from preclinical research with injected and oral THC and clinical observational studies of inhalation represent a major translational barrier, particularly for at-risk populations like post-menopausal women. As such, we hypothesize that THC exposure following osteoporosis will significantly impact bone health, exacerbating bone deficits induced by estrogen deficiency. To address this, we developed an inhalation-based rat model to determine whether chronic THC exposure mitigates or heightens the effects of ovariectomy on osteoporosis.

## 2. Methods

*Animals*: Adult, 26-week-old female Sprague–Dawley rats (Taconic, Rensselaer, NY, USA) were used in this study. All animals were single-housed in a temperature-controlled environment on a 12 h reverse light cycle (lights off 0600-1800). Food and water were provided ad libitum except during drug exposure. All experiments were conducted in compliance with the National Academy of Sciences Guide for the Care and Use of Laboratory Animals and approved by the University at Buffalo Institutional Animal Care and Use Committee.

**Figure 1:**
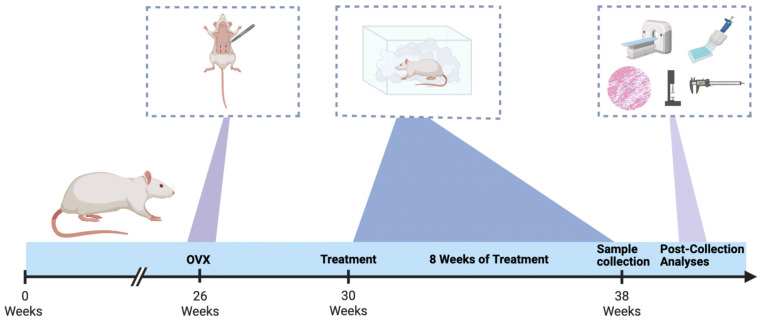
Experimental Timeline. Ovariectomy (OVX) surgeries were performed on 26-week-old Sprague–Dawley rats. This was followed by a 4-week period of post-menopausal-like bone loss. At 30 weeks rats began 8 weeks of drug treatment (THC or Air inhalation). After the drug treatment was completed, the animals were euthanized, and bone samples were harvested.

*Experimental Timeline:* After acclimation, the rats were randomized by body weight to the following 3 groups: (1) Sham surgery and Air treatment (*n* = 5); (2) Ovariectomy and Air treatment (*n* = 6); (3) Ovariectomy and THC treatment (*n* = 7). While OVX + Air represented osteoporosis under normal menopausal conditions, OVX + THC represented how THC treatment altered the course of post-menopausal osteoporosis. Sham-operated animals served as non-menopausal controls. Surgeries were performed when rats were 26 weeks of age. Treatment was timed to begin 4 weeks after surgery to allow for the development of post-menopausal-like hormonal changes and bone loss. At 30 weeks, rats began 8 weeks of drug treatment (THC or Air inhalation). After the drug treatment was completed, the animals were euthanized, and bone samples were harvested. Rats were weighed weekly throughout the study. A summary of the experimental timeline can be seen in Figure 1.

*Drug Treatment:* THC was obtained from the NIDA Drug Supply Program (North Bethesda, MD, USA) and prepared for inhalation in a 95% ethanol vehicle from a 200 mg/m stock solution. The inhalation procedure was adapted from a previously established protocol [45]. Briefly, animals were placed in a sealed inhalation chamber (2.5 gallons; 16.5 × 11.3 × 5.5 inches) and allowed a 5 min habituation period prior to exposure. THC (0.25 mL; 40 mg) was applied to steel pads, and the ethanol was fully evaporated before vaporization. Based on previous findings, 40 mg in our specific inhalation apparatus results in a dose of 0.76 mg/kg [45] in rodents. The human equivalent dose would be 0.11 mg/kg, which is within the human range of inhalation dose in humans (range, 0.01–0.5 mg/kg/d) [45,52]. The compound was vaporized using a Volcano vaporizer (Storz and Bickel, Tuttlingen, Germany) at heat setting 9 (approximately 226 °C), and the resulting vapor was collected in an 8 L plastic balloon. The vapor was then delivered into the airtight chamber via a fitted adaptor, and the animals were exposed for 10 min. All exposures were conducted under a fume hood, and animals were continuously monitored throughout the procedure. Following exposure, animals were returned to their home cages. Control animals underwent the same procedure but were exposed to Air only.

*Ovariectomy (OVX):* For surgery, a 4 × 4 cm area of the dorsal side of the rat was shaved and sterilely prepped. A 1–2 cm incision was made about 1 cm lateral of the midline, and the skin was blunt dissected. The fascia and muscle tissue were bluntly dissected to expose the abdominal cavity. Once the ovary and fallopian tube were identified, the ovary was isolated and ligated using 4-0 silk monofilament sutures twice, 2 mm apart, on the fallopian tube. The ligations were made as close to the ovary as possible while avoiding any ovarian tissue. The ovary was then removed, and the muscle layer was closed with 4-0 absorbable sutures. The skin was then stitched closed with 4-0 absorbable sutures. This procedure was repeated for the second ovary. Following surgery, rats were injected with carprofen (5 mg/kg) for 3 days post-op for pain management and Baytril (5 mg/kg) for 7 days post-op to prevent infection. For animals in the ShamSx + Air group, ovaries were exposed, not ligated and the incision was sutured closed like the experimental group. The purpose of sham surgeries was to serve as a control for the stress of surgery.

*Euthanasia and Tissue Collection:* After 8 weeks of drug treatment the rats were deeply anesthetized with 5% isoflurane and exsanguinated via cardiac puncture. The rear legs were then harvested. Right tibiae were isolated, soft tissues were removed, and the bones were incubated in 10% neutral buffered formalin (NBF) at 4 °C for 24 h. The NBF was then changed, and the bones were fixed for an additional 24 h. The bones were then transferred to 70% ethanol and stored at 4 °C until further processing. Left femurs were isolated, soft tissues were removed, and the bones were wrapped in gauze dampened in PBS and stored at −20 °C. Left tibias were isolated, soft tissues were removed, and the bones were immersed in 70% ethanol and stored at −20 °C. Whole blood was allowed to clot at room temperature for 45 min. Samples were then centrifuged at 604× *g* and 4 °C for 15 min, aliquoted, and stored at −80 °C until analysis.

*Caliper Measurements:* To assess longitudinal growth, left tibiae were measured with digital calipers (Mitutoyo, Aurora, IL, USA). Length was measured from the top of the tibial condyles to the bottom of the medial malleolus. Diameter was measured along the anterior–posterior (AP) and medial-lateral (ML) axes at the tibial plateau (proximal) and mid-diaphysis (mid) [53,54,55].

*Microcomputed Tomography:* Left femora were scanned to evaluate the microstructure and density of trabecular and cortical compartments using a uCT40 (Scanco, Brüttisellen, Switzerland) at a voltage of 55 kV, current of 145 uA, and resolution of 18 μm. Images were then reconstructed using uCT Tomography (Ver. 6.3-4, Scanco). Trabecular analyses were conducted for a metaphyseal region of interest (ROI) beginning 250 slices below the proximal epiphysis and continuing for an additional 115 slices (2.07 mm). Automated scripts were then used to calculate bone fraction (BV/TV), bone mineral density (BMD), trabecular number (Tb. N), trabecular thickness (Tb. Th), trabecular separation (Tb. Sp), and structural model index (SMI). Cortical analyses were conducted for an ROI comprising 100 slices (1.80 mm), centered at the femoral mid-diaphysis, and included cortical volume (Ct. V), periosteal volume (Ps. V), endocortical volume (Ec. V), cortical thickness (Ct. Th), bone mineral density (BMD), and polar moment of inertia (pMOI) [54,55,56,57,58].

*Biomechanical Analyses:* Following microCT analyses, the left femur underwent three-point bending to assess biomechanical integrity. Prior to testing, the femora were brought to room temperature and kept hydrated in saline. They were then placed in a custom-designed stainless steel loading jig with an outer span of 20 mm. A monotonic load to failure was applied along the AP axis at a rate of 20 mm/min, under displacement control, using a MTESTQuattro materials testing system equipped with a 1000 N load cell (Admet, Norwood, MA, USA). Load and displacement were sampled at 100 Hz using the MTESTQuattro software package (Version 3.13.01, Admet, Norwood, MA, USA). Ultimate force, stiffness, and energy to failure were calculated from force vs. displacement plots using a set of custom-written macros in Excel (Microsoft, Redmond, WA, USA) [53,58].

*Growth Plate Histology:* Growth plate height and morphology were assessed in right tibiae. Briefly, the specimens were decalcified in 5% formic acid (Sigma Aldrich, Burlington, MA, USA) for ~14 days, dehydrated in ethanol and xylenes, and embedded in paraffin. Coronal sections (5 μm) were then cut with a microtome (Leica, Hesse, Germany) and stained with Safranin O/Fast Green. To quantify growth plate height, digital images of the growth plates were acquired from 3 sections/rats at 200× magnification using an inverted microscope (Eclipse E800, Nikon, Shinagawa, Japan) equipped with a digital camera (Infinity3, Lumenera, ON, Canada) using Infinity Capture (Ver. 6.5.7, Lumenera). Growth plate height was then measured at three locations (medial, central, and lateral) for 3 sections per rat image using ImageJ (Ver 1.53) [55,56,57].

*ELISA Analysis:* Serum blood levels of estradiol (E2) were measured using ELISA assays (ELISA from MyBioSource, San Diego, CA, USA).

*Statistics:* Shapiro–Wilk tests were performed to assess normality, and subsequent comparisons were made using one-way ANOVA or Kruskal–Wallis tests if they passed or failed normality, respectively. Pairwise comparisons of parametric data were made using Tukey’s multiple comparisons tests to minimize type I error. Pairwise comparisons of non-parametric data were made using Dunn’s multiple comparisons tests. All analyses were performed using Prism (Ver 10.3.1, GraphPad, Boston, MA, USA) at an alpha of 0.05.

## 3. Results

**Figure 2:**
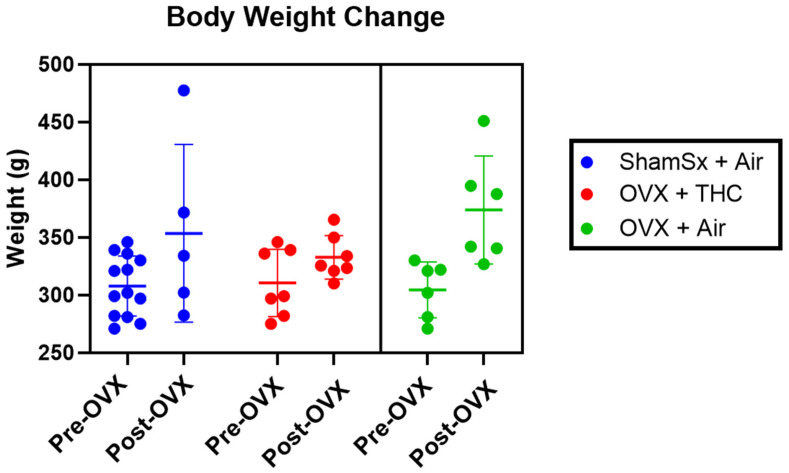
Body weights over time. Dot plots (mean line + SD) show body weights of ShamSx + Air (*n* = 5), OVX + THC (*n* = 7), and OVX + Air (*n* = 6) groups before surgery in grams. No significant difference between groups was found [F(2, 23) = 0.08476; *p* = 0.9190]. The right box and whisker plots show body weights of ShamSx + Air (*n* = 5), OVX + THC (*n* = 7), and OVX + Air (*n* = 6) groups at the end of the study in grams. No significant difference between groups was found [F(2, 15) = 1.113; *p* = 0.3542].

*Body Weight:* No significant differences in body weight were identified between Air and THC rats at the time of ovariectomy surgery [F(2, 23) = 0.08476; *p* = 0.9190] using one-way ANOVA (Figure 2). Similarly, one-way ANOVA comparing the body weights of all groups at the end of the study showed no significant difference between groups [F(2, 15) = 1.113; *p* = 0.3542]; Figure 2.

**Figure 3:**
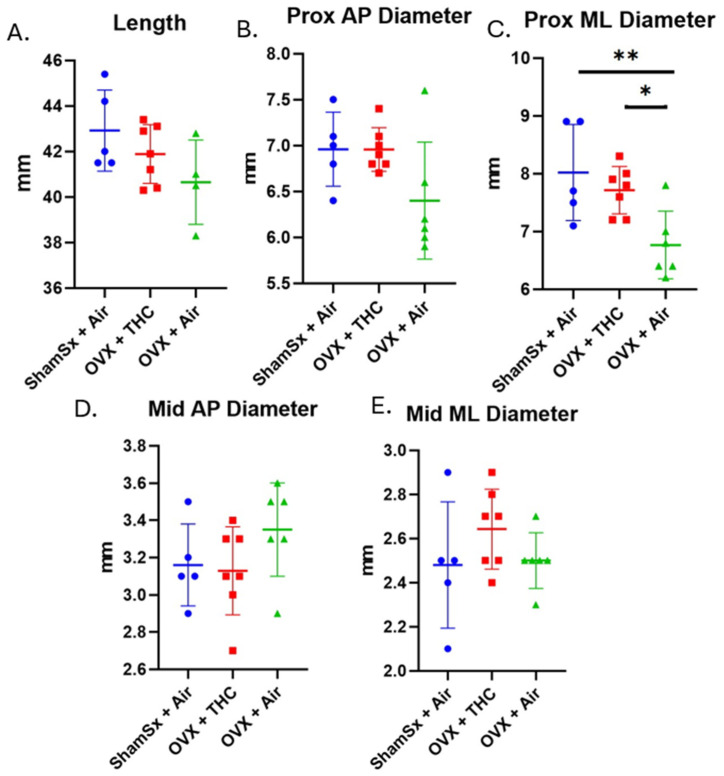
Caliper measurements. Dot plots (mean line + SD) showing femoral (**A**) length, (**B**) proximal medial-lateral diameter, (**C**) proximal anterior–posterior diameter, (**D**) mid-diaphysis medial-lateral diameter, (**E**) mid-diaphysis anterior–posterior diameter. Significant differences between treatment groups (ANOVA) are indicated by lines. * *p* < 0.05; ** *p* < 0.005.

*Caliper Measurements:* Analysis of bone length and width via caliper measurements revealed significant differences in proximal medial-lateral diameter [F(2, 15) = 6.687, *p* = 0.0084](Figure 3C). Specifically, when compared to ShamSx + Air rats, OVX + Air rats showed a significant decrease in proximal ML diameter (16%, *p* = 0.01; Figure 3C). Additionally, compared to OVX + Air rats, OVX + THC rats showed a significant decrease (12%, *p* = 0.03; Figure 3C). Length, proximal anterior–posterior, mid-diaphysis anterior–posterior and medial-lateral diameter measurements did not show any significant differences (Figure 3A,B,D,E).

### 3.1. MicroCT Results

**Figure 4:**
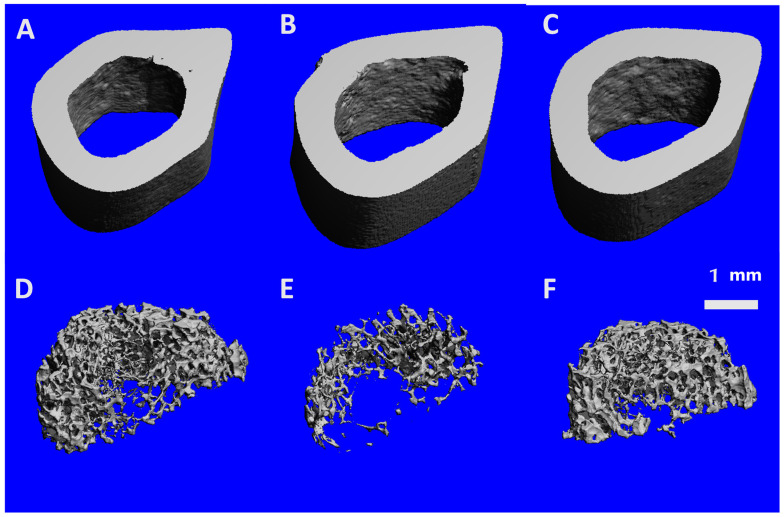
Surface-rendered microCT images. Representative series of femoral microCT images of mid-diaphyseal cortical regions of interest from (**A**) ShamSX + Air; (**B**) OVX + THC; (**C**) OVX + AIR; and proximal metaphyseal trabecular regions of interest from: (**D**) ShamSX + Air; (**E**) OVX + THC; (**F**) OVX + AIR. The scale bar represents 1 mm.

**Figure 5:**
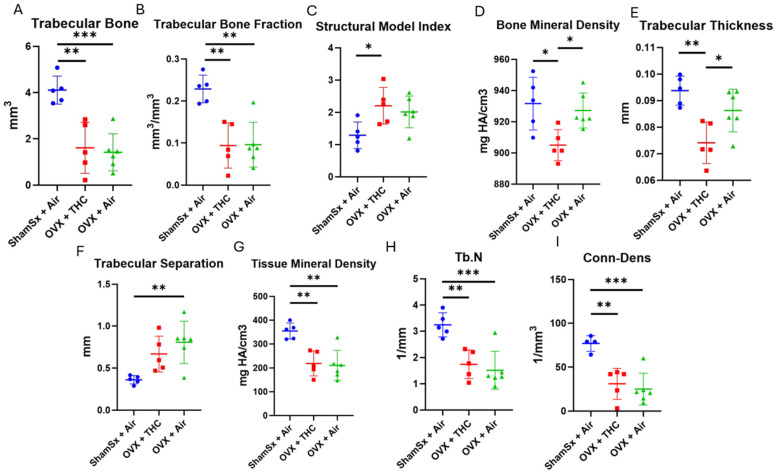
Trabecular microCT results. Dot plots (mean line + SD) showing trabecular microCT parameters from the proximal tibial metaphysis in ShamSx + Air, OVX + THC, and OVX +Air groups. Parametric results include (**A**) trabecular bone volume; (**B**) bone volume fraction; (**C**) structural model index; (**D**) volumetric bone mineral density; (**E**) trabecular thickness; (**F**) trabecular separation; (**G**) tissue mineral density. Non-parametric results include (**H**) trabecular number and (**I**) connective density. Significant differences between treatment groups (one-way ANOVA) are indicated by lines. * *p* < 0.05; ** *p* < 0.005; *** *p* < 0.001.

Analysis of femurs by microCT revealed a significant difference in various bone parameters, including proximal metaphyseal trabecular and mid-diaphyseal cortical regions of interest from each group (Figure 4). Data for connective density and trabecular number were found to be non-parametric using Shapiro–Wilk tests, and thus, subsequent analysis of these parameters was performed using Kruskal–Wallis tests. Significant differences were revealed in connective density (H(2) = 8.543; *p* = 0.0054) and trabecular bone number (H(2) = 9.347, *p* = 0.003; Figure 5H,I). Dunn’s multiple comparisons revealed a significant decrease in trabecular bone number (53%; *p* = 0.0099; Figure 5H) and connective density (77%; *p* = 0.0140; Figure 5I) of OVX + Air compared to ShamSx + Air rats.

**Figure 6:**
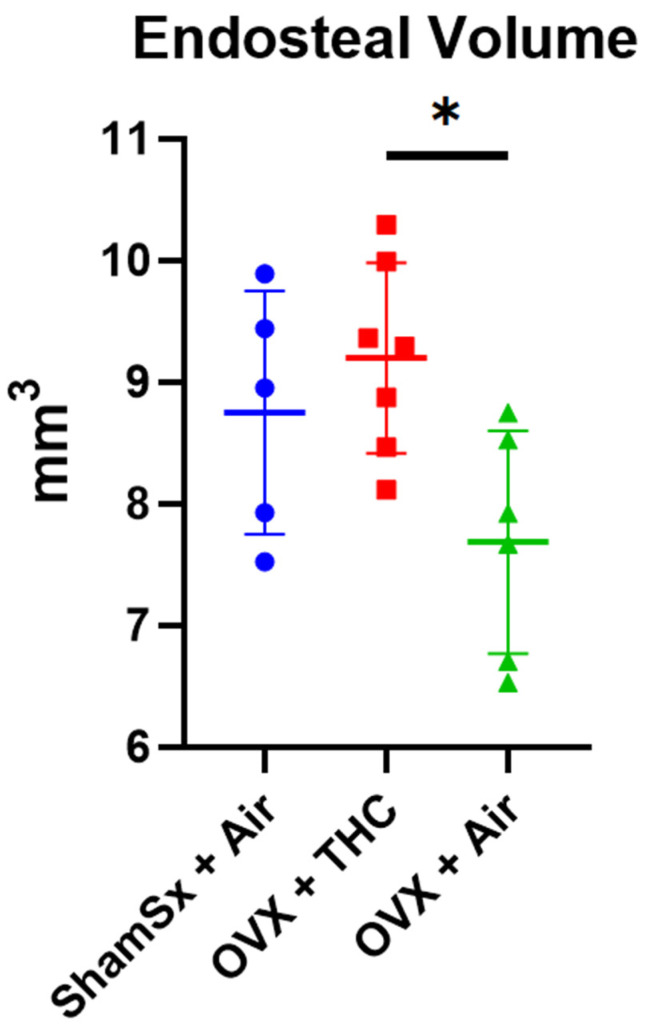
Cortical microCT results. Dot plots (mean line + SD) showing cortical microCT parameters from the proximal tibial metaphysis in ShamSx + Air, OVX + THC, and OVX + Air groups. Endosteal volume was significantly increased between the OVX + THC group and the OVX + Air group. Data was found to be parametric. Significant differences between treatment groups (Kruskal–Wallis) are indicated by lines. * *p* < 0.05.

All other parameters were found to be parametric using Shapiro–Wilk tests and were analyzed using one-way ANOVA. Of the measures analyzed by ANOVA, significant differences were found in bone volume [F(2, 13) = 15.95, *p* = 0.0003], bone volume fraction [F(2, 13) = 13.17, *p* = 0.0007], structural model index [F(2, 13) = 4.829, *p* = 0.0270], trabecular thickness [F(2, 13) = 9.365, *p* = 0.0030], trabecular spacing [F(2, 13) = 7.091, *p* = 0.0083], tissue mineral density [F(2, 13) = 12.61, *p* = 0.0009], bone mineral density [F(2, 13) = 6.206, *p* = 0.0128] (Figure 5A–G), and endosteal volume [F(2, 13) = 4.823, *p* = 0.0241] (Figure 6). Post hoc tests revealed that in OVX + THC rats compared to ShamSx + Air rats, a significant decrease was found in trabecular bone volume (61%; *p* = 0.0013; Figure 5A), bone volume fraction (59%; *p* = 0.0019; Figure 5B), trabecular thickness (21%; *p* = 0.0024; Figure 5E), tissue mineral density (38.35%; *p* = 0.0031; Figure 5G), and bone mineral density (2.86%; *p* = 0.0159; Figure 5D). A significant increase was identified in structural model index (71%; *p* = 0.0296; Figure 5C). In OVX + THC rats compared to OVX + Air rats, a significant decrease was revealed in trabecular thickness (14%; *p* = 0.0392; Figure 5E) and bone mineral density (2%; *p* = 0.0348; Figure 5D). In OVX + THC rats compared to OVX + Air rats, a significant increase was found in endosteal volume (20%; *p* = 0.0204; Figure 6). In OVX + Air rats compared to ShamSx + Air rats, a significant increase was found in bone volume (65%; *p* = 0.0005; Figure 5A), bone volume fraction (58%; *p* = 0.0015; Figure 5B), trabecular spacing (123%; *p* = 0.0068; Figure 5F), and tissue mineral density (41%; *p* = 0.0014; Figure 5G). MicroCT results are summarized in Table 1.

### 3.2. Biomechanics

All biomechanics data were found to be parametric using Shapiro–Wilk tests; thus, data was analyzed by one-way ANOVA. No significant differences were found in any measure. Measures included Energy to Failure [F(2, 15) = 1.890; *p* = 0.1853], Stiffness [F(2, 15) = 2.025; *p* = 0.1665], Ultimate Force [F(2, 15) = 1.350, *p* = 0.2889], Failure Force [F(2, 15) = 1.515, *p* = 0.2516], or Yield Force [F(2, 15) = 0.2117; *p* = 0.8116] (see Table 2).

**Figure 7:**
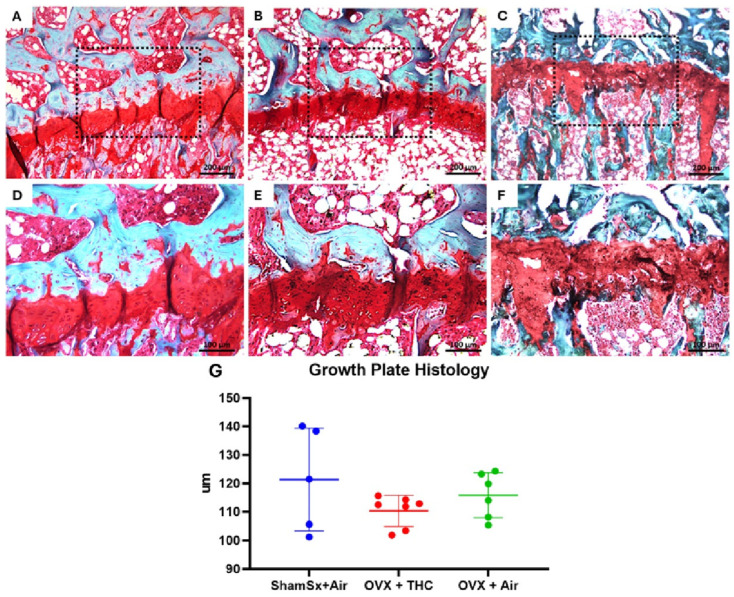
Growth plate histology. Photomicrographs of proximal tibial growth plates after stain with Safranin O/Fast Green.: (**A**) ShamSx + Air; (**B**) OVX + THC; (**C**) OVX + Air at low magnification (100×, scale bars = 200 µm); (**D**) ShamSx + Air; (**E**) OVX + THC; (**F**) OVX + Air at high magnification (200×, scale bars = 100 µm); (**G**) dot plots (mean line + SD) showing mean growth plate height (line) for each group. No significant differences were found between groups.

No significant differences in growth plate size were found via one-way ANOVA [F(2, 15) = 1.493; *p* = 0.2562; Figure 7). Compared to Air + ShamSx, OVX + THC resulted in a 9% decrease, while OVX + Air resulted in a 5% decrease in mean growth plate size but these differences were not significant (Air + Sham Sx vs. OVX + THC; *p* = 0.2302), (Air + Sham Sx vs. OVX + Air; *p* = 0.6891).

### 3.3. E2 ELISA Results: ELISA Immunoassay

**Figure 8:**
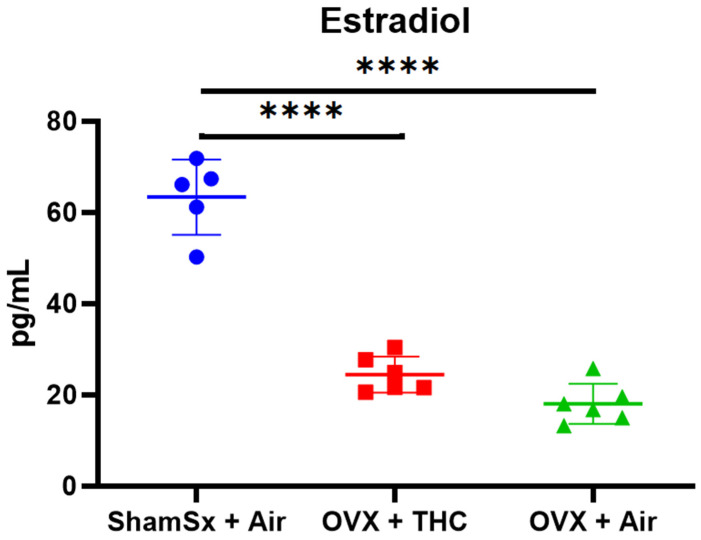
Estradiol (E2) ELISA results: Bar plots show mean serum E2 levels in OVX + THC, OVX + Air, and ShamSx + Air following 8 weeks of drug treatment. One-way ANOVA revealed a significant difference between groups [F(2, 14) = 100.2; *p* < 0.0001]. Tukey’s multiple comparisons test revealed significant differences between the OVX + THC and ShamSx + OVX groups (*p* < 0.0001) and the OVX + Air and ShamSx + Air groups (*p* < 0.0001). **** *p* < 0.0001.

One-way ANOVA revealed a significant difference in serum estradiol concentration [F(2, 14) = 100.2; *p* < 0.0001; Figure 8]. Compared to control animals (ShamSx + Air), OVX + THC animals showed a 61% decrease in mean serum estradiol, while OVX + Air animals showed a 71% decrease. Tukey’s multiple comparisons test revealed significant differences between the OVX + THC and ShamSx + OVX groups (*p* < 0.0001) and the OVX + Air and ShamSx + Air groups (*p* < 0.0001).

## 4. Discussion

While prior studies have examined the effects of cannabis exposure on bone, few have investigated chronic inhaled THC specifically in the context of estrogen deficiency for a clinically relevant at-risk population, particularly with respect to trabecular microarchitecture and bone remodeling outcomes. Therefore, we developed an inhalation-based rat model to determine whether chronic THC exposure mitigates or heightens the effects of ovariectomy on osteoporosis. The trabecular deterioration observed in the OVX + Air rats, including reduced bone volume fraction, increased trabecular spacing, decreased tissue mineral density, and loss of trabecular number and connectivity density, is consistent with previous research patterns of a well-characterized estrogen deficiency-induced bone loss due to accelerated osteoclastic resorption leading to perforation and disappearance of trabeculae [59,60,61]. Interestingly, THC exposure compounded these osteoporotic changes, as shown by OVX + THC animals exhibiting further reductions in trabecular thickness and bone mineral density beyond those attributed to ovariectomy alone. This additive’s deleterious effect aligns with previous in vitro research evidence that THC exerts dose-dependent stimulatory effects on osteoclastic bone resorption at lower concentrations, as well as with clinical data that associates heavy cannabis use with a lower bone mineral density and elevated bone turnover markers [36,48,62]. It is likely the mechanism involves THC activation of cannabinoid receptors expressed on osteoblasts and osteoclasts, CB1 and CB2. Previous research has found CB1 receptor signaling to regulate ovariectomy-induced bone loss, and pharmacological antagonism of both receptors has been osteoprotective in ovariectomy models [63,64]. In contrast to the widespread trabecular changes observed, cortical bone was largely spared, with the only significant finding being increased endosteal volume in OVX + THC rats compared to OVX + Air controls. This compartment-specific pattern is consistent with previous literature and the established understanding that estrogen deficiency preferentially targets trabecular microarchitecture, while the cortical changes manifest primarily as endocortical resorption and bone marrow cavity expansion, rather than gross cortical thinning [65,66]. The selective increase in endosteal volume observed with THC exposure to ovariectomized animals suggests that THC may potentiate endocortical resorption when in an estrogen-deficient state, a finding that warrants further investigation given the importance of bone integrity for fracture resistance.

Building on these findings, the present study suggests that chronic THC inhalation following the onset of the estrogen deficiency does not attenuate osteoporotic bone loss and may instead further impair select aspects of trabecular bone microarchitecture. While OVX alone produced the expected reductions in trabecular integrity, chronic THC exposure was associated with additional deficits, particularly in trabecular thickness and bone mineral density when compared to OVX controls. These changes were also accompanied by reductions in trabecular number and connectivity density, further contributing to deterioration of trabecular network structure. The observed increase in structural model index indicates a shift toward a more rod-like trabecular structure, which is generally considered mechanically weaker and more susceptible to fracture [67]. Together, these results suggest that THC may contribute to a more fragile trabecular microarchitectural phenotype in the context of osteoporosis.

The mechanisms underlying these effects may involve dysregulation of the ECS, which plays a critical role in maintaining the balance between bone formation and bone resorption [68]. Osteoblasts and osteoclasts express cannabinoid receptors, with CB1 signaling associated with the bone remodeling processes and CB2 generally linked to anti-resorptive and protective effects [63,69]. Importantly, estrogen was shown to regulate ECS activity, as estradiol increases CB1 and CB2 receptor expression and levels of the endocannabinoid AEA [70], while also inhibiting osteoclast activity through CB2-mediated mechanisms [71]. In the context of estrogen deficiency, this regulatory balance is disrupted, promoting increased osteoclast activity and bone resorption. This is supported by the ELISA analysis, which demonstrated a significant reduction in serum estradiol levels in both OVX groups compared to sham controls, with OVX + THC animals showing a 61% decrease and OVX + Air animals showing a 71% decrease. No significant differences were observed between OVX + THC and OVX + Air animals, indicating that the THC exposure did not alter circulating estradiol levels.

Under these conditions, chronic THC exposure may further dysregulate ECS signaling. Although THC acts as a partial agonist of both CB1 and CB2 receptors, it exhibits greater affinity for CB1 receptors [20]. Prolonged THC exposure has also been shown to induce receptor desensitization and impair downstream signaling pathways [72,73]. Both CB1 and CB2 receptors have been implicated in bone remodeling by influencing osteoblast differentiation and osteoclast activity [20,63,69]. Within bone tissue, CB1 receptors expressed on osteoblasts and sympathetic nerve terminals have been associated with regulation of bone formation and osteoclast activity, with excessive CB1 signaling linked to increased bone resorption and bone loss [63,74]. In contrast, CB2 receptors are generally associated with anti-resorptive effects through suppression of osteoclast differentiation and activity (Idris 2008 [69] and Ofek 2006 [75]).

Consistent with this, inhibition of CB1 signaling was shown to attenuate osteoporosis-related bone loss [63,76,77], whereas CB2 agonism has been associated with increased osteoblast activity and reduced osteoclast activity [34,75,78,79]. Disruption of the balance of osteoblast and osteoclast activity in favor of osteoclast-mediated resorption would therefore be expected to promote bone loss and reduced bone formation [63,72]. When combined with estrogen deficiency, which independently promotes osteoclast-driven bone loss [80], these effects may act synergistically to exacerbate deterioration in trabecular bone microarchitecture. Given THC’s greater affinity for CB1 receptors, the trabecular deterioration observed in the present study may therefore reflect predominantly CB1-mediated effects on bone remodeling. However, because the downstream mechanisms of CB1 and CB2 signaling in osteoporosis remain incompletely understood, these interpretations should be considered cautiously. These hypothesized mechanisms of action should be interrogated by future studies.

The selective impact of THC on trabecular bone observed in this study is consistent with the known biological properties. Trabecular bone has a higher surface area, greater metabolic activity, and a more rapid turnover compared to cortical bone, making it particularly sensitive to hormonal and physiological changes [81]. As such, early osteoporotic changes are typically most pronounced in trabecular regions, with cortical alterations and biomechanical deficits emerging later in disease progression [82]. This likely explains why significant differences were observed in trabecular parameters, while cortical structure and biomechanical strength remained largely unchanged. This may also reflect the relatively short interval between OVX induction and tissue collection, as well as the age at which osteoporosis was induced, both of which can influence the extent to which structural changes translate into measurable biomechanical deficits.

Despite clear microarchitectural deterioration, no significant differences were detected in biomechanical properties. This may reflect the relatively short duration of THC exposure or the possibility that the bone’s internal structure worsens before changes in overall strength can be detected, which is consistent with the greater sensitivity of trabecular bone to early osteoporotic changes. Similarly, the increase in endosteal volume observed in OVX + THC animals suggests enhanced resorption along the inner cortical surface, which may represent an early indicator of cortical remodeling that has not yet translated into functional deficits.

Previous clinical and preclinical studies examining cannabis and bone health have yielded mixed findings, and other drugs of abuse have routinely been shown to alter bone health and development [53,54,55,83,84], supporting a broader role for substance exposure in disrupting skeletal homeostasis. One clinical study linked chronic cannabis use to reduced BMD and increased fracture risk [48], while a preclinical study suggested potential therapeutic effects for specific cannabinoids [85]. Differences in cannabinoid composition, dosing, route of administration, and timing relative to disease onset likely contribute to the inconsistency of these studies. The present findings highlight that in the context of established estrogen deficiency, chronic THC exposure alone does not provide skeletal benefit and may instead worsen bone microarchitecture.

Translationally, these results suggest that THC use in populations at risk for osteoporosis, particularly post-menopausal individuals, may negatively impact bone quality by altering remodeling processes rather than improving skeletal integrity. These findings have important implications given the increasing prevalence of cannabis use, particularly among populations at risk for osteoporosis, and underscore the need for further research into the skeletal effects of cannabinoids.

## 5. Limitations

Several limitations should be considered. The sample size was relatively small, which may limit statistical power for detecting differences in certain outcomes. Type I errors are a potential concern due to the number of parameters analyzed. Additionally, only a single dose and duration of THC exposure were examined. Future studies should investigate dose-dependent effects, and longer exposure periods and incorporate detailed molecular analyses of bone turnover and ECS signaling to further elucidate the mechanisms underlying these effects. Further, our results only capture the effect of THC on menopausal osteoporosis and likely would not translate to male rodents or patients with osteoporosis.

## 6. Conclusions

In our model of post-menopausal osteoporosis utilizing ovariectomy to induce an estrogen-deficient state, administration of THC vapor following the procedure exacerbated the negative impacts of estrogen deficiency on multiple metrics of trabecular microarchitecture. Changes in the trabecular compartment are concerning, as early osteoporotic changes are most pronounced in trabecular bone. Notable changes in THC-treated rats included increased endosteal volume (20%; *p* < 0.05), decreased trabecular bone mineral density (2%; *p* < 0.005), and decreased trabecular thickness (14%; *p* < 0.05). Our methodology reflects chronic use of a low dose of THC and suggests that even mild use of THC may negatively affect post-menopausal women with respect to bone health. Future research should test the effects of higher doses and attempt to elucidate the molecular mechanisms underlying microarchitectural changes observed in the current study.

## Figures and Tables

**Table 1:** Micro CT analyses. The groups were compared by ANOVA; multiple comparisons were made between groups using Tukey’s HSD to correct for type I error. Resulting *p*-values are shown. ^a^ indicates data was parametrically assessed by Shapiro–Wilk tests. ^b^ indicates data was non-parametric.

		ShamSx + Air		OVX + THC		OVX + Air		*p* = Value	Significant Pairwise Comparisons with Tukey’s HSD
		Average	StDev	Average	StDev	Average	StDev		
Trabecular	Total Volume	18.08	2.19	15.51	3.65	14.55	1.55	*p* = 0.1035 ^a^	-
	Bone Volume (mm^3^)	4.10	0.60	1.61	1.10	1.42	0.80	*p* = 0.0003 ^a^	ShamSx + Air vs. OVX + THC, ShamSx + Air vs. OVX + Air
	Bone Volume Fraction	0.23	0.03	0.09	0.05	0.10	0.05	*p* = 0.0007 ^a^	ShamSx + Air vs. OVX + THC, ShamSx + Air vs. OVX + Air
	Connectivity Density (1/mm^3^)	107.13	68.07	31.03	17.68	25.01	18.04	*p* = 0.0054 ^b^	ShamSx + Air vs. OVX + Air
	Structural Model Index	1.29	0.41	2.20	0.57	2.01	0.49	*p* = 0.0270 ^a^	ShamSx + Air vs. OVX + THC, ShamSx + Air vs. OVX + Air
	Trabecular Number (1/mm)	3.24	0.46	1.74	0.54	1.51	0.72	*p* = 0.0031 ^b^	ShamSx + Air vs. OVX + Air
	Trabecular Thickness (mm)	0.09	0.01	0.07	0.01	0.09	0.01	*p* = 0.0030 ^a^	ShamSx + Air vs. OVX + THC, OVX +THC vs. OVX + Air
	Trabecular Spacing (mm)	0.36	0.05	0.67	0.21	0.81	0.25	*p* = 0.0083 ^a^	ShamSx + Air vs. OVX + Air
	Tissue Mineral Density	354.31	33.65	218.44	51.82	210.46	62.72	*p* = 0.0009 ^a^	ShamSx + Air vs. OVX + THC, ShamSx + Air vs. OVX + Air
	BMD (mg HA/cm^3^)	931.61	16.92	904.98	9.91	927.17	11.20	*p* = 0.0128 ^a^	ShamSx + Air vs. OVX + THC, OVX +THC vs. OVX + Air
Cortical	Bone Volume (mm^3^)	15.51	1.89	15.30	1.36	14.02	1.25	*p* = 0.2120 ^a^	-
	Cortical Thickness (mm)	0.74	0.05	0.71	0.04	0.70	0.05	*p* = 0.4539 ^a^	-
	BMD (mg HA/cm^3^)	1189.81	11.65	1184.09	14.53	1200.23	8.32	*p* = 0.0822 ^a^	-
	Endosteal Volume (mm^3^)	8.75	1.00	9.20	0.78	7.68	0.91	*p* = 0.0241 ^a^	OVX + THC vs. OVX + Air
	Periosteal Volume (mm^3^)	24.21	2.88	24.47	1.86	21.68	1.65	*p* = 0.0687 ^a^	-
	Polar Moment of Inertia (mm^4^)	17.00	3.92	17.34	2.86	13.61	2.01	*p* = 0.0820 ^a^	-
	Imax (mm^4^)	10.49	2.44	11.00	2.19	8.40	1.34	*p* = 0.0876 ^a^	-
	Imin (mm^4^)	6.51	1.52	6.34	0.85	5.21	0.88	*p* = 0.1417 ^b^	-

**Table 2:** Biomechanical analyses. The groups were compared by ANOVA. Resulting *p*-values are shown.

	ShamSx + Air	OVX + THC	OVX + Air	*p*-Value
	Mean ± SD	Mean ± SD	Mean ± SD	
Energy to Failure (mJ)	59.31 ± 7.74	79.06 ± 25.1	72.39 ± 14	*p* = 0.1853
Stiffness (N/mm)	209.37 ± 1 3.23	195.4 ± 29.34	185.79 ± 63.77	*p* = 0.1665
Ultimate Force (N)	141.68 ± 8.33	156.98 ± 26.72	149.61 ± 35.33	*p* = 0.2889
Failure Force (N)	141.6 ± 8.43	156.98 ± 26.72	147.82 ± 35.84	*p* = 0.2516
Yield Force (N)	124.14 ± 4.56	131.77 ± 12.12	135.03 ± 39.03	*p* = 0.8116

## Data Availability

The original contributions presented in this study are included in the article. Further inquiries can be directed to the corresponding author.
